# Surface Properties of the Polyethylene Terephthalate (PET) Substrate Modified with the Phospholipid-Polypeptide-Antioxidant Films: Design of Functional Biocoatings

**DOI:** 10.3390/pharmaceutics14122815

**Published:** 2022-12-15

**Authors:** Klaudia Szafran, Małgorzata Jurak, Robert Mroczka, Agnieszka Ewa Wiącek

**Affiliations:** 1 Department of Interfacial Phenomena, Institute of Chemical Sciences, Faculty of Chemistry, Maria Curie-Skłodowska University, 20-031 Lublin, Poland; 2 Laboratory of X-ray Optics, Department of Chemistry, Institute of Biological Sciences, Faculty of Medicine, The John Paul II Catholic University of Lublin, 20-708 Lublin, Poland

**Keywords:** polyethylene terephthalate, cyclosporine A, lauryl gallate, AFM, TOF-SIMS, wettability

## Abstract

Surface properties of polyethylene terephthalate (PET) coated with the ternary monolayers of the phospholipid 1,2-dioleoyl-*sn*-glycero-3-phosphocholine (DOPC), the immunosuppressant cyclosporine A (CsA), and the antioxidant lauryl gallate (LG) were examined. The films were deposited, by means of the Langmuir–Blodgett (LB) technique, on activated by air low temperature plasma PET plates (PET_air_). Their topography and surface chemistry were determined with the help of atomic force microscopy (AFM) and time-of-flight secondary ion mass spectrometry (TOF-SIMS), respectively, while wettability was evaluated by the contact angle measurements. Then, the surface free energy and its components were calculated from the Lifshitz–van der Waals/Acid–Base (LWAB) approach. The AFM imaging showed that the Langmuir monolayers were transferred effectively and yielded smoothing of the PET_air_ surface. Mass spectrometry confirmed compatibility of the quantitative and qualitative compositions of the monolayers before and after the transfer onto the substrate. Moreover, the molecular arrangement in the LB films and possible mechanisms of DOPC-CsA-LG interactions were determined. The wettability studies provided information on the type and magnitude of the interactions that can occur between the biocoatings and the liquids imitating different environments. It was found that the changes from open to closed conformation of CsA molecules are driven by the hydrophobic environment ensured by the surrounding DOPC and LG molecules. This process is of significance to drug delivery where the CsA molecules can be released directly from the biomaterial surface by passive diffusion. The obtained results showed that the chosen techniques are complementary for the characterization of the molecular organization of multicomponent LB films at the polymer substrate as well as for designing biocompatible coatings with precisely defined wettability.

## 1. Introduction

**Figure 1:**
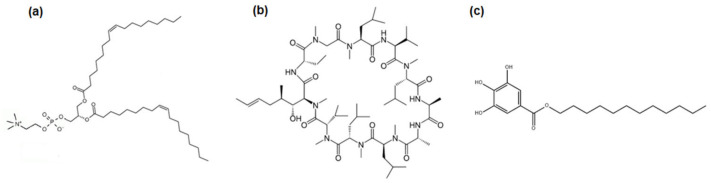
Structure of 1,2-dioleoyl-*sn*-glycero-3-phosphocholine, DOPC (**a**), cyclosporine A, CsA (**b**), and lauryl gallate, LG (**c**).

This paper is focused on designing modern coatings for biomaterials with polymer which can be used as prostheses or their elements. Apart from the function of replacing living tissues, they can also deliver drugs effectively to the desirable place, eliminating the problem of drug bioavailability or therapy side effects. One of the most commonly used polymers in tissue engineering as artificial ligament transplants [1,2] and coating for blood vessels’ metallic stents, is polyethylene terephthalate (PET) [3,4,5]. Its usage is possibly owing to good mechanical properties as well as corrosion resistance and large strength to weight ratio [6]. However, due to hydrophobicity, chemical inertness, poor biocompatibility, the possibility of hydrolytic degradation, and thrombosis, its usage is confined and can lead to unfavorable biological reactions [3]. To counteract these adverse properties, its surface can be modified by plasma treatment [7] and bioactive coatings [3]. Plasma action demands the addition of new active species which can form functional groups [8]. This modification consists of changing only the outer surface without changing its bulk properties [9]. Due to this process the polymer surface has a more hydrophilic character [10], and it is possible to deposit layers of biological substances effectively. Among such substances are phospholipids (PLs) that can significantly improve the compatibility of the implant, changing the body’s response to a positive one and avoiding a rejection of the artificial material [3,11]. Such PLs, particularly those that have a choline group in their structure, are building components of biological cell membranes [12]. The use of one of them, 1,2-dioleoyl-*sn*-glycero-3-phosphocholine (DOPC, Figure 1a), provides the fluid state of natural membranes. It also makes it possible to get to know and better understand the interaction mechanisms between living tissue and drugs following implantation. Moreover, the phospholipid films of this type can be a platform for introducing active substances or drugs directly onto the implant surface.

The drug of interest to this study is cyclosporine A (CsA, Figure 1b) which has strong immunosuppressive properties and is used in medicine mainly to avoid graft rejection, but also to treat psoriasis [13], dry eye disease [14], and rheumatoid arthritis [15]. From a chemical point of view, CsA is a cyclic polypeptide with a high molecular weight (1203 Da) which makes it highly hydrophobic. Its small affinity for water and physiological fluids [16] causes serious problems in the effectiveness of the therapy, which consists of inhibiting T-lymphocytes and cytokine suppression [17,18]. Therefore, in common oral administration, only a slight effectiveness (10%) can be achieved. Hence, the introduction of CsA directly onto the implant surface seems to be a suitable treatment alternative. On the other hand, the administration of CsA is associated with the occurrence of undesirable side effects which include abnormalities in the functioning of the kidneys [19], heart [20], and circulatory system [21]. The negative effect of CsA on the functioning of the human body is mainly related to the release of free radicals which leads to damage of the biological membranes of organs. To minimize and/or eliminate the above effects, it is necessary to develop an appropriate composition of the implant coating that will improve the drug bioavailability [22,23]. One way to prevent membrane damage could be the parallel use of lauryl gallate (LG, Figure 1c).

LG is an antioxidant derived from gallic acid, characterized by possessing the greatest antioxidant activity in the group of alkyl derivatives [24,25]. LG can protect membranes as it is able to trap free radicals [26] produced by an immunosuppressant, and, additionally, is characterized by possessing antimicrobial properties [27,28]. 

Therefore, the graft surface’s morphology (selection of coating components and their proportions), and its wettability [23], and the way the membrane’s building components (PLs) interact with drugs (CsA) are essential for cell adhesion, proliferation, and differentiation. The response of the human body to implanted material, caused mainly by the action of the immune system, is inflammation and/or clots formation. Such processes can lead to implant rejection. Therefore, the current research on the surface modification of implants plays an important role in improving their biocompatibility. Surface morphology, the kind of functional groups, implant wettability (hydrophilic-hydrophobic nature, surface free energy), and topography are very important parameters influencing the adaptation of the implanted materials [29]. 

Different types of groups introduced onto the implant surface, for example by means of plasma modification [30,31,32,33], strongly influence the adhesion and multiplication of platelets or other cells [34]. In turn, the topography of the implant surface significantly affects the interactions between it and the surrounding cells. The wettability of the material determines the interactions with proteins, which also impacts on the binding and multiplication of platelets during the contact of the implant with human tissues [29]. Accordingly, a determination of thermodynamic functions [35], such as surface free energy and its components, can be helpful in estimating the type of interactions between the membrane-implant surface and its surroundings. The examination of morphology is an important point in the research to improve the PET biocompatibility and to design novel biocoatings for polymer-made implants that can also act as drug-controlled release systems.

The goal of our studies is to gain insight into the properties of mixed DOPC-CsA-LG (assigned as DCL) monolayers with varying molar fractions of LG and a constant DOPC-CsA (assigned as DC) molar ratio of 1:1 after transferring them onto activated by air plasma PET substrate (PET_air_). Previously, the use of single DOPC, CsA, and LG, and mixed (binary and ternary) monolayers deposited on mica and gold substrates, were tested [36]. The novelty of these studies lays in the transfer onto activated surfaces of polymer PET, a material that is used in the tissue engineering. The deposition of the examined monolayers onto the PET polymer support offers a development of knowledge about its topography, molecular orientation (spatial arrangement), chemical composition, wettability determined by the atomic force microscopy (AFM), time-of-flight secondary ion mass spectrometry (TOF-SIMS), and contact angle measurements, respectively. In addition, the determination of the surface free energy and its components for this type of Langmuir–Blodgett films has not been described in the literature so far. It is extremely important to gain knowledge of the physicochemical properties of the modified surfaces to design compatible biocoatings which can be of great potential application in medicine.

## 2. Materials and Methods

### 2.1. Solution Preparation

The proper amount of 1,2-dioleoyl-*sn*-glycero-3-phosphocholine (DOPC, ≥99%, Sigma, St. Louis, MO, USA), cyclosporine A (CsA, ≥99%, Alfa Aesar, Kandel, Germany), and lauryl gallate (LG, ≥99%, Aldrich, St. Louis, MO, USA) was dissolved in the chloroform:methanol (4:1, *v*:*v*) mixture to obtain the final concentration equal to 1 mg mL^−1^. Chloroform (99.8%) and methanol (≥99.9%) were purchased from Macron Fine Chemicals and from Fluka^TM^, respectively. Then, by mixing the basic solutions, binary (DOPC-CsA with the 1:1 molar ratio) and ternary (DOPC-CsA-LG with the LG molar fraction ($\chi_{LG}$) equal to 0.25, 0.50, 0.75) ones were obtained. 

### 2.2. PET Plates Cleaning

The PET plates (20 × 20 × 3 mm^3^), cut from the commercially available material (Bayer Material Science, Leverkusen, Germany), were cleaned with methanol (99.8%, Avantor Performance Materials S.A., Gliwice, Poland) and deionized Milli-Q water (from Milli-Q purification system, Millipore, Burlington, MA, USA) of resistivity 18.2 MΩ cm, in the ultrasonic bath (UM4, Unima, Olsztyn, Poland) for 15 min. Then, the plates were dried in the exsiccator for 24 h at room temperature (21° ± 1°). 

### 2.3. Plasma Modification

The cleaned PET plates were placed in the plasma generator chamber (Plasma type system, Diener Electronic, Ebhausen, Germany) for 1 min to activate their surface with the air low temperature and low pressure (0.20 mbar) plasma. The power of the air plasma was 460 W, and the plasma process ran with a continuous gas flow of 22 sccm. After the one-minute action the plates were removed for further modifications.

### 2.4. Langmuir Monolayer Preparation

Once cleaned with acetone (99.5%, Avantor Performance Materials S.A) and methanol, and rinsed three times with Milli-Q water, the Langmuir–Blodgett KSV 2000 Standard trough (KSV, Helsinki, Finland) was used to deposit single DOPC, CsA, LG, or mixed monolayers onto the air plasma activated PET plates. The surface tension was measured by means of the Wilhelmy plate with a 0.1 mN m^−1^ accuracy. Knowing the size of the trough and the concentration of the solutions, the proper volume (15–50 μL) of solution was placed onto the water subphase using a microsyringe (Hamilton-Bonaduz, Bonaduz, Switzerland). Then, the system was left for 10 min in order to evaporate the solvent. In the next stage, symmetrical compression using the automatically moved barriers, at a rate of 20 mm min^−1^, was performed. At the same time, the surface pressure-area per molecule (π−A) isotherm was registered. 

### 2.5. Brewster Angle Microscopy (BAM) Analysis

Morphology of the monolayers was examined by means of the Brewster angle microscope nanofilm_ultrabam (Accurion GmbH, Goettingen, Germany) with a lateral resolution of 2 μm. The ultrabam, equipped with the solid-state 50 mW laser emitting *p*-polarized light at 658 nm wavelength, was applied (at Brewster angle of 53.2°) to take BAM images. Additionally, the monolayer relative thickness at the air/water interface (d) was estimated. First, before placing the solution on the water subphase, the camera calibration was made. Thereby, the plot of the gray level as a function of incidence angle was obtained as the minimum of the parabolic fit. After the solution spread and the solvent evaporation, the monolayer was compressed. Simultaneously, the gray scale data were converted into the reflectivity (R) and then into the relative film thickness (d) according to Equation (1) in compliance with the single-layer optical model [37]:

(1) $$R=\frac{I_{r}}{I_{0}}={\left(\pi \frac{d}{\lambda }\right)}^{2}\frac{{\left(n_{1}^{2}-n_{2}^{2}-1+\frac{n_{2}^{2}}{n_{1}^{2}}\right)}^{2}}{1+n_{2}^{2}}$$

where: $I_{0}$ means the incident intensity, $I_{r}$ is the reflected intensity, and $n_{1}$ and $n_{2}$ indicate the refractive indices of the film and pure subphase, respectively; λ is the wavelength of the incident light.

### 2.6. Langmuir–Blodgett (LB) Film Preparation

In the next stage, to deposit the Langmuir monolayer onto the activated by air low temperature plasma PET substrate the thin film was compressed to a surface pressure of 10 mN m^−1^ (the presence of all monolayer components is ensured at this surface pressure value). After reaching the given surface pressure the PET substrate was withdrawn from the subphase through the compressed film with a barrier speed of 5 mm min^−1^. When prepared, the LB films deposited on the PET_air_ substrate were dried to remove traces of water and then stored in the dark glass exsiccator for 24 h to protect them from moisture-induced restructuring and light-induced damages before the AFM, TOF-SIMS, and CA measurements.

The transfer ratio (TR) as an indicator of the efficiency of Langmuir monolayer transfer from the liquid phase to the polymer substrate and of the LB film quality was calculated according to Equation (2).

(2) $$TR=\frac{\Delta A_{m}}{A_{s}}$$

where: $\Delta A_{m}$ denotes decrease in the monolayer surface area on the subphase and $A_{s}$ means substrate coated area. 

Determination of the transfer ratio value required providing the correction for the loss of molecules during the transfer of the monolayer due to the instability of the films (Supporting information in [36]). 

### 2.7. Atomic Force Microscopy (AFM) Analysis

An atomic force microscope (5600LS AFM, Aglient Technologies, Palo Alto, CA, USA) was used to investigate the nanostructure of the Langmuir–Blodgett monolayers on the polymer support in three dimensions. The tests were carried out in a non-contact mode (tip radius < 7 nm, resonance frequency 330 kHz) with a resolution of 256 × 256 and a scanning area of 20 × 20 μm^2^. The measurements were performed in at least three randomly selected locations on the sample. The post processing data analysis was made using the Probe Image Processor (SPIP) software v. 5.1.4 (Image Metrology, Hørsholm, Denmark). 

### 2.8. Time of Flight Secondary Ion Mass Spectrometry (TOF-SIMS) Analysis

Before the measurements, the samples were placed in the ultra-high vacuum (*p* < 10^−9^ mbar) chamber of the TOF-SIMS.5 instrument (ION-TOF GmbH, Münster, Germany). Subsequently, the TOF-SIMS spectra were obtained. The primary ion source of Bi^+^ was used at 30 keV and corresponded to 1.0 pA primary beam current in the spectrometry mode. The scanning area of the secondary ions was 200 × 200 μm^2^ with 256 × 256 pixels (1 shot/pixel) in the spectrometry mode. All the measurements were performed in the positive static mode (dose < 1 × 10^12^ ions cm^−2^). To neutralize the charge left on the surface, an electron flood gun (20 eV) and the surface potential (U = −360 V) were applied. The post-processing data analysis was conducted using SurfaceLab 6.7 software (ION-TOF) and Origin 2019 (OriginLab, Northampton, MA, USA). The spectra calibration was applied by means of the positions of $CH_{3}^{+}$, $C_{2}H_{3}^{+}$, and $C_{2}H_{5}^{+}$ fragments. All fragment intensities were normalized to the total intensity.

### 2.9. Contact Angle (CA) Measurements

A contact angle measuring apparatus (DGD ADR, GBX S.A.R.L, Romans-sur-Isére, France), coupled with a camera and an automatically tilting table controlled by Windrop++ software, was used to determine the contact angles. For each experiment, three test liquids with a well-known surface tension: Milli-Q water, formamide (99.5%, Acrōs Organics, Geel, Belgium), and diiodomethane (99%, Sigma-Aldrich) were applied. For every modified PET substrate and every test liquid, the same volume of liquid (6 μL) was placed. Then, using the computer software, the advancing contact angle was measured from the settled droplet shape. Moreover, the measurements of the contact angle values of the probe liquids were taken within 60 s. The values of water, formamide, and diiodomethane contact angles were used for further calculation of the surface free energy and its components.

### 2.10. Surface Free Energy (SFE) and Its Components Calculations

The Lifshitz–van der Waals/Acid–Base (LWAB, [38,39,40]) model was used to calculate the total surface free energy and its components. Based on this approach the work of adhesion ($W_{A}^{a}$) is dependent on the interactions which appear between the solid (s) and the liquid (l) at the interface (Equation (3)).

(3) $$W_{A}^{a}=\gamma_{l}\left(1+\cos\theta_{A}\right)=2\sqrt{\gamma_{s}^{LW}\gamma_{l}^{LW}}+2\sqrt{\gamma_{s}^{+}\gamma_{l}^{-}}+2\sqrt{\gamma_{s}^{-}\gamma_{l}^{+}}$$

The authors of the LWAB approach defined the total surface free energy as the sum of apolar $\gamma_{s}^{LW}$ and polar $\gamma_{s}^{AB}$ interactions (Equations (4) and (5)).

(4) $$\gamma_{s}^{tot}=\gamma_{s}^{LW}+\gamma_{s}^{AB}$$

(5) $$\gamma_{s}^{AB}=2\sqrt{\gamma_{s}^{-}\gamma_{s}^{+}}$$

where: $\theta_{A}$ is the average advancing contact angle, $\gamma_{s}^{tot}$ is the total surface free energy, $\gamma_{s}^{LW}$ is the Lifshitz–van der Waals component, $\gamma_{s}^{AB}$ is the Lewis acid–base component, $\gamma_{s/l}^{-}$ is the electron-donor parameter of solid or liquid, and $\gamma_{s/l}^{+}$ is the electron-acceptor parameter of solid or liquid. 

To calculate the $\gamma_{s}^{tot}$ value there is a need to solve three equations of Equation (3) with three unknown quantities.

## 3. Results and Discussion

The Langmuir and Langmuir–Blodgett techniques are very useful for the design and characterization of biocompatible implant coatings which can deliver drugs directly to the desired (implanted) place in a human organism simultaneously. Before the thin film is deposited on the solid substrate, it is important to investigate the monolayer properties of the liquid subphase. In a previous paper, the thermodynamic parameters (excess area per molecule, excess and total Gibbs energy of mixing), as well as the changes in the surface potential and the apparent dipole moment, were determined [41]. This allowed for the comprehensive characterization of the monolayer organization and the interactions between the molecules forming thin films. All examined monolayers at the air/water interface were found to be in a liquid state (L) which was proved by the compression modulus values [41]. In this study, the morphology and thickness of the Langmuir films, before the transference at a surface pressure of 10 mN m^−1^, were determined by means of the Brewster angle microscopy (BAM) and compared with the topography, root mean square roughness ($S_{q}$), and height profiles obtained for the LB films with the help of AFM. The representative parameters are presented in Table 1. Beyond the surface morphology (BAM) and topography (AFM), the organization of the monolayer molecules (TOF-SIMS) and the surface wettability (CA) were investigated. For all quantities, the experimental/numerical error was expressed as the standard deviation estimated from at least three measured/calculated values. For the AFM and TOF-SIMS measurements, three randomly chosen areas at the modified PET substrate were examined.

### 3.1. BAM Analysis and Monolayer Transfer Efficiency

**Figure 2:**
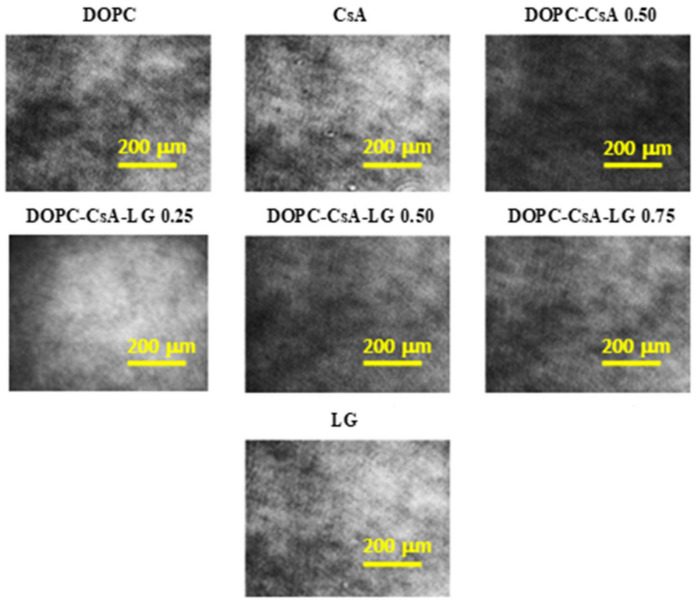
BAM images taken for the single (DOPC, CsA, LG), binary (DOPC-CsA 0.50), and ternary (DOPC-CsA-LG 0.25, 0.50, 0.75) monolayers at 10 mN m^−1^ on the water subphase.

In order to investigate the morphology of all studied monolayers on the water subphase, BAM images were taken (Figure 2). During the compression no phase separation was observed in the entire range of surface pressures, i.e., homogeneous films were obtained. Figure 2 presents only the representative BAM images taken at a surface pressure of 10 mN m^−1^. This is in agreement with the miscibility of DOPC-CsA-LG monolayer components described previously [41].

In the next stage, on the basis of the reflectivity measurements the relative thickness (d) of the tested monolayers at the air/water interface was determined (Equation (1)). The average thickness values were obtained in the range of 2–2.5 nm (Table 1). Among the single monolayers, the LG film is characterized by having the largest thickness value. The reason for this can be the presence of saturated hydrocarbon chains (Figure 1c) which promote changes in the molecule’s orientation to a more vertical one during the compression. In contrast, a slightly smaller thickness was obtained for the single DOPC film. This can result from the kink caused by double *cis*-bound in the hydrocarbon chains (Figure 1a). The unsaturated bounds induce a greater inclination of the chains, therefore, the distances between the molecules increase, and thickness value is reduced. The single CsA monolayer is marked by the smallest thickness value since this is a cyclic molecule capable of different conformation formations (Figure 1b). In the case of the mixed (binary and ternary) monolayers, they obtained comparable thickness values (Table 1). 

To get information about the effectiveness of the deposition on the plasma activated PET substrates, the TR values were acquired. A TR value equal to 1 confirms that one layer of molecules was transferred onto the solid substrate [42]. The TR values determined for most of the investigated monolayers were close to 1 proving that these films were successfully transferred to the PET surface by the LB technique and that high quality LB monolayers were prepared (Table 1). The exception was the single DOPC monolayer which was characterized by having the biggest TR (1.6). This could be related to the conformation of the DOPC monolayer to the roughness of the PET surface which resulted in a higher TR. A slightly lower value was obtained for the binary DOPC-CsA 0.50 monolayer. The repulsive interactions between the molecules forming this monolayer [41] can contribute to the transfer of a smaller amount of molecules to the solid polymer substrate. The presence of the third component—LG in the mixed DOPC-CsA-LG monolayers—led to the changes in the type of interactions, from repulsion to attraction. Therefore, bigger TR values were obtained (Table 1). Such strong attraction interactions can promote excessive molecules transfer onto PET_air_.

**Table 1:** Thickness (d) and transfer ratio (TR) values for the indicated monolayers obtained at the air/water interface as well as $S_{q}$ roughness parameter gained by means of AFM for the modified PET substrates.

Monolayer	d	TR	$S_{q}(\mathrm{nm})$
DOPC	2.2 ± 0.1	1.6 ± 0.1	0.91 ± 0.19
CsA	2.0 ± 0.1	0.9 ± 0.1	1.63 ± 0.16
DC 0.50	2.1 ± 0.1	0.8 ± 0.2	0.82 ± 0.05
DCL 0.25	2.5 ± 0.1	1.0 ± 0.2	0.89 ± 0.13
DCL 0.50	2.1 ± 0.1	1.0 ± 0.3	0.92 ± 0.21
DCL 0.75	2.3 ± 0.1	1.0 ± 0.3	1.02 ± 0.33
LG	2.5 ± 0.1	0.9 ± 0.1	0.76 ± 0.04

### 3.2. AFM Analysis

Atomic force microscopy (AFM) is widely used in the study of structure in the nanoscale of various surfaces. Its high resolution and ability to detect very small forces, together with the possibility of working in an aqueous environment or physiological fluids, make it a good technique for studying biological systems [43] including biomaterials with a potential application as organ substitutes [42,44,45,46]. In the latter aspect, roughness is of particular importance as it determines the adhesion of coatings to solid supports, surface wettability, the adhesion and multiplication of cells [29] on the implanted materials, interactions with proteins [47,48,49], and therefore their biocompatibility. Thus, many attempts are made to ensure the appropriate surface roughness for these specific applications. 

**Figure 3:**
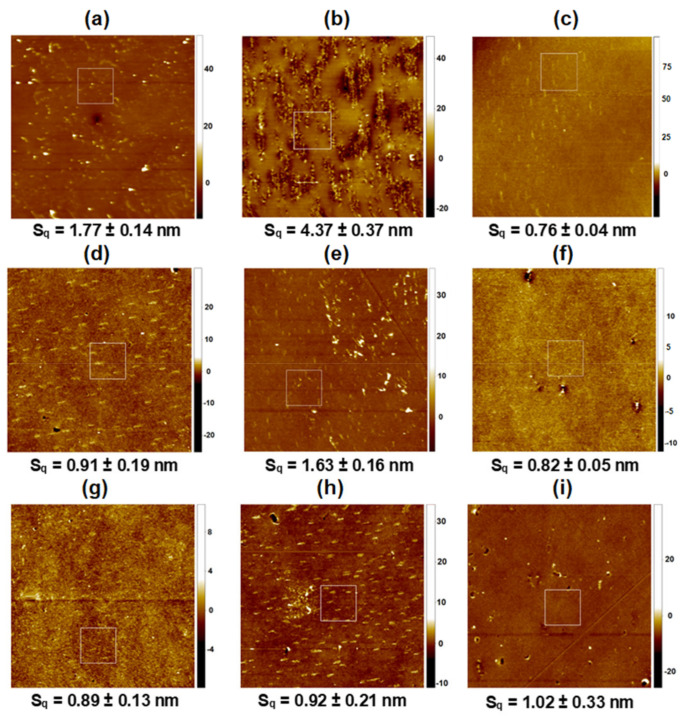
AFM micrographs for 20 μm × 20 μm scanned areas: PET (**a**), PET_air_ (**b**), PET_air_/LG (**c**), PET_air_/DOPC (**d**), PET_air_/CsA (**e**), PET_air_/DOPC-CsA 0.50 (**f**), PET_air_/DOPC-CsA-LG 0.25 (**g**), PET_air_/DOPC-CsA-LG 0.50 (**h**), PET_air_/DOPC-CsA-LG 0.75 (**i**). Z scale: nm.

**Figure 4:**
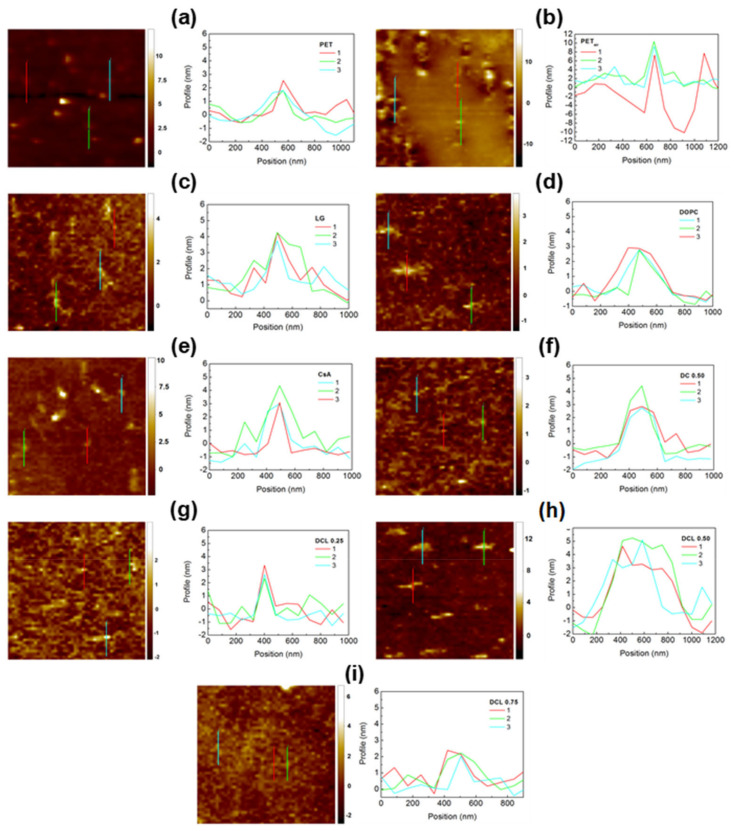
AFM zoomed micrographs and profiles obtained for: PET (**a**), PET_air_ (**b**), PET_air_/LG (**c**), PET_air_/DOPC (**d**), PET_air_/CsA (**e**), PET_air_/DOPC-CsA 0.50 (**f**), PET_air_/DOPC-CsA-LG 0.25 (**g**), PET_air_/DOPC-CsA-LG 0.50 (**h**), PET_air_/DOPC-CsA-LG 0.75 (**i**). Z scale: nm.

In this contribution, the AFM technique was used to determine the topography and surface roughness of the PET substrate, modified with plasma (PET_air_) and covered with monomolecular film composed of DOPC, CsA, and/or LG molecules of varying compositions. Figure 3 shows the AFM micrographs for the 20 × 20 μm^2^ scanned area with the marked zoomed area (with the Root Mean Square, $S_{q}$, roughness parameter) while Figure 4 shows the extracted zoomed area (left column) and the profiles corresponding to the marked lines (right column). The values of $S_{q}$ roughness parameter were determined according to Equation (6).

(6) $$S_{q}=\sqrt{\frac{1}{MN}\overset{M-1}{\underset{k=0}{\sum }}\overset{N-1}{\underset{l=0}{\sum }}{\left[z\left(x_{k},y_{l}\right)\right]}^{2}}$$

where: x and y are the coordinates, z is the perpendicular deviation from the ideally smooth surface, M is the number of points in the x direction, N is the number of points in the y direction, k and  l are the measuring points.

$S_{q}$ was chosen to analyze the surface roughness of the modified PET substrates because it is more sensitive to high peaks and deep valleys. The arithmetic average roughness parameter ($S_{a}$) can not define morphology of the examined surface sufficiently because the profiles with different shapes but the same arithmetic height of peaks and depth are presented as the same value of $S_{a}$ [50].

Although the BAM images (Figure 2) did not reveal the domain structures, the AFM micrographs demonstrated them (Figure 3). This is because the lateral resolution of the Brewster angle microscope is 2 μm while the atomic force microscope gives the information in the nanoscale. The formation of such structures can result from the reorganization of molecules accompanying their transfer from the liquid phase (pure water) to the solid support (activated PET surface) and its roughness. 

The $S_{q}$ parameter for the unmodified PET substrate is equal to almost 2 nm (Figure 3a) proving that the surface is smooth. This is consistent with the previously published data [9,51]. Its surface is characterized by numerous round protrusions about 2.3 nm height with a diameter of almost 380 nm (Figure 4a). Although the PET surface has a low level of roughness, some researchers found that, after implanting stents coated with this polymer, unfavorable immune responses occurred [52]. Therefore, there is a need to modify the PET’s properties. One of the methods of surface modification is activation by low temperature plasma. Its action more than doubles the surface roughness ($S_{q}$~4 nm, Figure 3b) in comparison with the unmodified PET. The PET_air_ is largely scratched while the significant part remains relatively smooth (flat patches and longitudinal domains). Similar results were obtained by Pandiyaraj et al. [10]. The authors claimed that the air plasma action results in the addition of new functional groups containing oxygen and nitrogen (-OH, C-O, O=C-O, C=O, N-CO-N) to the PET substrate [10,51,53]. Furthermore, their occurrence entailed an increase in the PET surface roughness in comparison with the untreated one. Similarly, other researchers [53,54,55] showed that the oxygen-containing plasma treatment introduced new polar functional groups with carbon and oxygen which enhances the PET surface roughness. This is related to the polymer chain breaking and adding oxygen from plasma [50,55,56].

It is hard to determine the surface roughness of the freshly plasma-treated PET substrate due to its exposure to the air atmosphere before the AFM measurements. It seems that during the exposure to the air, the chemical processes taking place in the outermost surface layer can induce significant protrusions and morphological changes (Figure 3b). On the other hand, all examined layers were deposited immediately after the plasma treatment. Hence, the PET substrate was protected against air exposure and roughness changes. Thus, the surface chemistry of PET_air_ is completely different from that of unmodified PET.

After the monolayer deposition onto the activated PET substrate, smoother surfaces are obtained (Table 1). The smoothest surface of the tested ones ($S_{q}$~0.8 nm, Figure 3c) is gained after transferring the 2.5 nm thick LG monolayer from the air/water interface to the polymer surface with a TR value close to 1. In this case, the surface waviness with a height up to 6 nm is visible at an angle of 45° to the substrate withdrawal direction (profile line not shown). The part of the layer with greater thickness consists of numerous, small longitudinal domains located parallel to the withdrawal direction with a height equal to about 2.7 nm, and a width almost 170 nm (Figure 4c). The non-uniform thickness of the LG layer over the entire surface and the slightly larger size of the domains in comparison with unmodified PET substrate is obtained. The reorganization of the structure indicates weak interactions of LG with the substrate, which can be the reason for its removal from the PET_air_ surface during the measurements taken with TOF-SIMS (see Section 3.3).

Slightly larger surface roughness is obtained for PET_air_ modified with the single DOPC layer (~1 nm, Figure 3d). This can be induced by an excess of DOPC molecules deposited during the transference process (TR = 1.6, Table 1). The examined surface demonstrates characteristic domains located perpendicular to the PET withdrawal direction (Figure 4d) with the dimensions: height of 3.0 nm, width of nearly 450 nm. Since the height of protrusions is about 1 nm larger than for the PET substrate (Figure 4a), this suggests that specifically adsorbed domains which are 1.5 monolayer thick (thickness ~3 nm) can be formed on the PET protrusions while outside them the single DOPC monolayer, characterized by a better arrangement, is deposited. This observation correlates strictly with the transfer ratio value which is much higher than unity (TR = 1.6, Table 1) indicating that more than one layer is transferred onto the PET_air_ plate. 

The smoothing process was achieved to a lesser extent after the transfer of the single CsA monolayer (Figure 3e). The protrusions’ height increases by about 2 nm (in comparison to PET) while the diameter remains unchanged. The domains’ formation is due to the specific interactions of the CsA molecules with the functional groups of the activated PET substrate which provoke the reorganization within the monolayer. These interactions can be proved by the surface free energy components, particularly by the changes in the electron-donor and electron-acceptor parameters (Section 3.4). Additionally, Pandiyaraj et al. proved that after the plasma modification the immobilization of insulin and heparin onto the plasma activated PET surface was obtained due to the presence of the new -C=O and C-N groups [10]. Thus, in our case, a similar process can occur. Moreover, the researchers reported that changes in the peaks’ size, related to the aliphatic bonds in the chain, also evidenced the formation of new interactions [10].

Around the protrusion, a densely packed monolayer of CsA remains. This is in agreement with its stiffness (compared to DOPC and LG), determined by packing the cyclic rings. This is revealed in the relatively high value of the compression modulus (66 mN m^−1^) at the transfer pressure of 10 mN m^−1^ (48 mN m^−1^ and 30 mN m^−1^ for DOPC and LG, respectively) [41]. The obtained transfer value (0.9 ± 0.1) also confirms the monolayer formation on the polymer substrate (Table 1). Moreover, high protrusions on the right side of the micrograph are visible. This can suggest significant agglomeration in the vertical direction of CsA molecules. The confirmation of this phenomenon is presented in the height (23 nm) and width (500 nm) of the protrusions in this region. These significantly larger values unequivocally prove the local deposition of more CsA molecules.

The two-component DOPC-CsA 0.50 layer is characterized by the intermediate roughness ($S_{q}$~0.8 nm, Figure 3f) between those of the single DOPC and CsA layers, which is due to the smaller packing of the monolayer regarding that of CsA (compression modulus equal to 53 mN m^−1^ [41]). The addition of CsA to the DOPC molecules conduces a smoothing of the surface with respect to PET_air_/CsA. Taking into account the repulsive nature of interactions between both compounds, expressed by the positive values of the excess Gibbs energy of mixing [41], one can claim that the CsA molecules surrounded by the DOPC ones form a more homogeneous film on the PET_air_ surface. The mixing of the CsA and DOPC molecules to form a binary monolayer impedes the creation of DOPC domains thereby the CsA aggregation (Figure 3f). There occur two regions within the remaining area: the more densely packed monolayer (brighter areas) and the more loosely packed layer (darker color). Moreover, the surface protrusions are almost completely eliminated (Figure 3f). The levelling effect is probably determined by the preferential deposition of DOPC-CsA on the flat surface instead of the protrusions on the PET_air_ substrate.

Furthermore, the addition of LG does not affect the surface roughness significantly (Figure 3g–i). In the group of ternary monolayers, the comparable values of the surface roughness parameter are obtained. This fact can be ascribed to the similar physical phase of these films [41] as well as almost the same transfer ratio and monolayer thickness values (Table 1).

After deposition of the ternary DOPC-CsA-LG 0.25 monolayer the surface topography (Figure 2g) is similar to that of the binary DOPC-CsA 0.50 layer while the non-uniformity of the height profile distribution (waviness) is slightly higher, reaching 1.5 nm for DOPC-CsA-LG 0.25 and 1.2 nm for DOPC-CsA 0.50. Significant changes in topography are observed after the transfer of the ternary DOPC-CsA-LG 0.50 monomolecular film (Figure 3h). There are visible characteristic domains with the following dimensions: height of 4 nm and width of about 340 nm. The latter size was determined from the greater number of the domains’ sizes, while the most representative ones are shown in Figure 4h and demonstrate a greater similarity to the single DOPC domains. Moreover, in the middle-left side of this micrograph, close to the square box, the agglomeration of the molecules is visible (Figure 3h). A similar phenomenon occurs for the single CsA monolayer (Figure 3e). After increasing the LG molar fraction to 0.75, all domains disappear, and a rather smooth layer is formed (Figure 3i). A slight surface cover differentiation, and numerous scratches (uncovered areas), are visible.

This can be caused by the reorganization of the monolayer molecules on the PET substrate after the deposition process. Additionally, the LG molecules are tilted towards the solid substrate due to the head-to-tail mismatch, i.e., there is a big disproportion between the size of the polar head group and the hydrocarbon chain (Figure 1c). Therefore, the thin layer can not be densely packed with molecules located perpendicularly to the substrate surface. In this way, fragmentation can happen more easily (see Section 3.3). Additionally, analogous films characterized by the smallest surface roughness were obtained after their transfer to the gold substrate [36]. Thus, clearly indicating the significant influence of the type of substrate on the coating roughness [36,42].

To get more insight into the arrangement of the molecules forming the indicated monolayers a time-of-flight-secondary mass spectrometry was applied, and the obtained results are described in the next chapter.

### 3.3. TOF-SIMS Analysis

**Figure 5:**
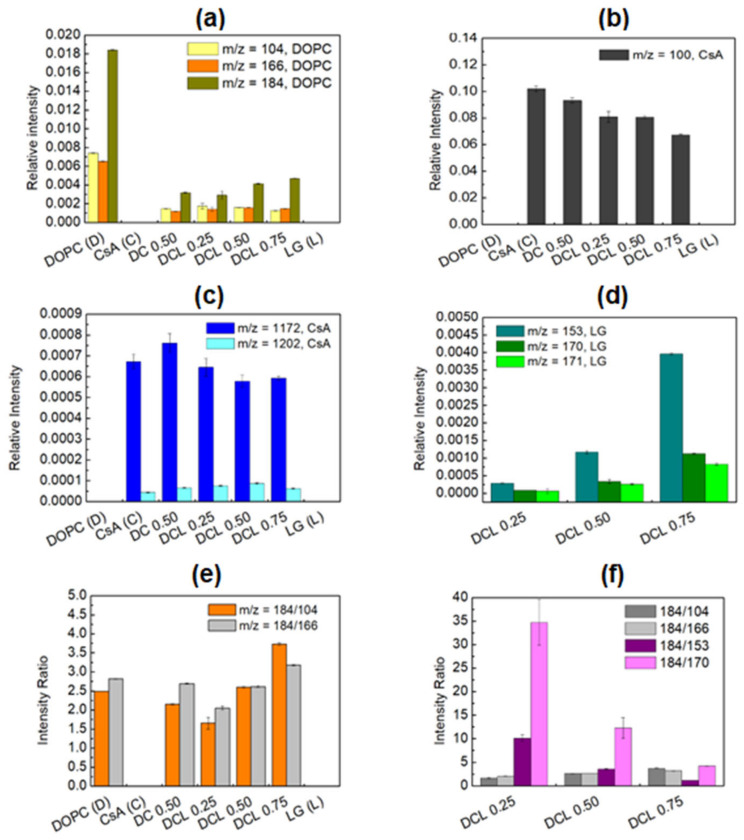
Distribution of 1,2-dioleoyl-*sn*-glycero-3-phosphocholine *m*/*z* = 104, 166, and 184 (phosphocholine) fragments (**a**), cyclosporine *m*/*z* = 100 fragment (**b**), cyclosporine *m*/*z* = 1172, 1202 fragments (**c**), lauryl gallate *m*/*z* = 153, 170, 171 fragments (**d**), intensity ratio of DOPC to DOPC fragments (**e**) intensity ratio of DOPC to DOPC and DOPC to LG fragments (**f**) in single, binary, and ternary monolayers deposited onto PET_air_.

**Figure 6:**
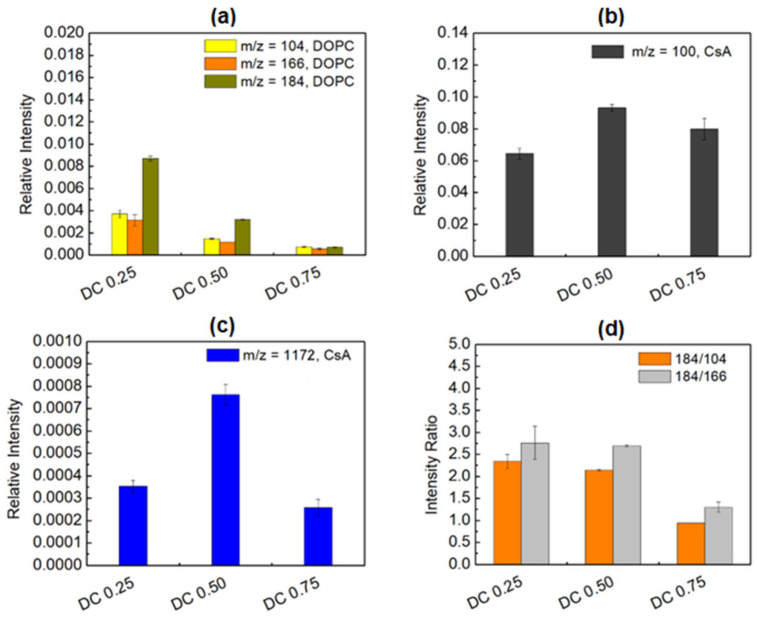
Distribution of 1,2-dioleoyl-*sn*-glycero-3-phosphocholine *m*/*z* = 104, 166, and 184 (phosphocholine) fragments (**a**), cyclosporine (C_6_H_14_N)^+^ fragment (**b**), cyclosporine *m*/*z* = 1172 fragment (**c**), intensity ratio of DOPC to DOPC fragments (**d**) in the binary DOPC-CsA monolayers with different molar fractions of CsA deposited onto PET_air_.

The single DOPC, CsA, LG, binary DOPC-CsA, and ternary DOPC-CsA-LG ($\chi_{LG}$ = 0.25, 0.50, 0.75) Langmuir monolayers deposited onto the activated by air low temperature plasma PET substrate were examined by means of the TOF-SIMS technique. The use of mass spectrometry obtains better insight into the interactions between the components of thin films as well as allowing for the determination of compound distribution [57]. The chemical structure and fragmentation pathways of the DOPC, CsA, and LG molecules with the assigned fragments identified in the TOF-SIMS spectra can be found elsewhere [58,59,60]. The most characteristic positive fragments identified in the TOF-SIMS mass spectra for the measured samples are listed in Table 2, while Figure 5 and Figure 6 show the average relative intensity values with the standard deviations representing the experimental error for the pure DOPC, CsA, LG, binary DOPC-CsA, and ternary DOPC-CsA-LG films at the LG molar fractions of 0.25, 0.50, and 0.75. 

As can be seen, the molecular ions of the single DOPC and LG layers are not found. A molecular ion is defined here as a positively charged molecule formed by the removal of one electron from a molecule as a result of electron beam action. Its mass is equal to the molecular mass of the examined molecule, while the pseudomolecular ion is yielded by the subtraction of two oxygen atoms from the molecular ion. The occurrence of a molecular ion can be an indicator of monolayer ordering strictly connected with the arrangement/tilting of the molecules. The lack of molecular ions can be caused by a few reasons. The first thing to consider is possible molecular disorder in the monolayer. The DOPC layer deposited onto the PET_air_ substrate is disordered due to the fluid nature of the monolayers (liquid expanded phase [41]). The presence of unsaturated bonds in the DOPC structure determines the small ability of forming densely packed and well-ordered monolayers by its molecules. To verify that disordering in the monolayer caused by unsaturated bonds is responsible for the lack of the molecular ions, the similar phospholipid consisting of saturated hydrocarbon chains 1,2-dipalmitoyl-*sn*-glycero-3-phosphocholine (DPPC) was transferred onto the solid support, forming a well-packed and ordered film. The molecular ion of this phospholipid in the TOF-SIMS spectra was identified [58]. Taking this into account, the conclusion is as follows: after activation of the PET substrate by the air plasma, the PC heads attach to the polymer support but the structure of chains prevents the formation of a very densely packed, well-ordered single DOPC monolayer as it was obtained for DPPC. This is probably related to the steric hindrance of bended unsaturated hydrocarbon chains.

**Table 2:** The most characteristic positive fragments of DOPC, LG, and CsA in the TOF-SIMS mass spectra.

Assignment	*m*/*z*	Identification	References
(C_5_H_14_NO)^+^	104	DOPC	[58]
(C_5_H_13_PO_3_N)^+^	166	DOPC	[58]
(C_5_H_15_NPO_4_)^+^	184	DOPC	[58]
(C_7_H_5_O_4_)^+^	153	LG	[58]
(C_7_H_6_O_5_)^+^	170	LG	[58]
(C_7_H_7_O_5_)^+^	171	LG	[58]
(C_6_H_14_N)^+^	100	CsA	[58]
(C_61_H_107_N_10_O_12_)^+^	1172	CsA pseudomolecular ion	[60]
(C_62_H_112_N_11_O_12_)^+^ (CsA + H)^+^	1202	CsA molecular ion	[60]

On the other hand, the molecular ion was identified previously for both phospholipids (DPPC and DOPC) in the TOF-SIMS mass spectra [58] for the monolayers deposited on the mica [59]. The atomically smooth mica promotes the creation of a more energetically homogeneous substrate with a large number of active canters that allows for the development of a more tightly packed monolayer. This indicates that the distance between the phosphocholine heads of DOPC is reduced. Moreover, the structure of the DOPC monolayer on mica was additionally examined by the AFM studies. For this reason, the existence of the molecular ion is a widely accepted indicator of the well-organized monolayer in which the molecules show a certain order. Additionally, the molecular ion for the single LG layer was also detected on the mica substrate [58]. This indicates the strong influence of the underlying substrate on monolayer organization.

Another important parameter to consider here is the surface pressure of the transference. In this contribution, a transfer surface pressure of 10 mN m^−1^ was applied, which ensured a more fluid state of monolayers than at a surface pressure of 35 mN m^−1^. Due to this fact it can be said that this is the primary factor determining the lack of molecular ions in the single DOPC and LG films. Hence, as was reported in our recent paper [36], both DOPC and LG molecules can be tilted towards the solid support after their deposition onto it.

The most intensive signals from various parts of the phosphocholine polar head *m*/*z* = 104 (C_5_H_14_NO)^+^, *m*/*z* = 166 (C_5_H_13_PO_3_N)^+^, *m*/*z* = 184 (C_5_H_15_NPO_4_)^+^ demonstrate a very strong intensity for the single DOPC layer (Figure 5a) which corresponds to the literature data [58,61,62,63]. For the single CsA monolayer the most prominent fragments of CsA *m*/*z* = 100 (C_6_H_14_N)^+^, (Figure 5b), pseudomolecular *m*/*z* = 1172 (C_61_H_107_N_10_O_12_)^+^, and molecular ion *m*/*z* = 1202 (C_62_H_112_N_11_O_12_)^+^ (Figure 5c), are identified, compliant with our previous publication [60]. The existence of the CsA molecular ion in the TOF-SIMS spectra was observed previously [60] for the layers deposited on the rough PEEK substrate. Identification of the molecular ion of CsA in our experiment on the plasma treated PET with a significantly smoother surface than that of PEEK strongly proves that the CsA molecule demonstrates a very high affinity for different surfaces. Furthermore, affirmation of the CsA molecular ion can be an indicator of a highly packed monolayer. Additionally, the pseudomolecular ion of CsA gives a significantly greater yield than the molecular ion (Figure 5c).

After mixing the CsA and DOPC molecules, the intensity of characteristic moieties for DOPC and CsA is changed. A slightly greater intensity of the pseudomolecular ion (*m*/*z* = 1172) is observed in comparison to the single CsA layer (Figure 5c). This suggests that the presence of DOPC molecules changes the orientation of the CsA molecules to be more perpendicular and, in consequence, the intensity of the pseudomolecular ion increases. A similar observation is generally found for the phospholipids molecules where the molecular ion is identified [58]. Moreover, the CsA amount in the DOPC-CsA layer decreases slightly in comparison with the one-component CsA film, as the intensity of the most intensive CsA ion ((C_6_H_14_N)^+^) decreases (Figure 5b). This is an obvious consequence of the presence of a smaller amount of CsA molecules in the mixed Langmuir monolayer.

The intensity of all the characteristic fragments of the phosphocholine head decreases about 6-times after mixing with CsA (DOPC-CsA 0.50, Figure 5a) while the intensity of the cyclosporine fragment (C_6_H_14_N^+^) only ca. 10% (Figure 5b). The above results clearly confirm that the molecules of both components are present in the LB film. On the other hand, this large intensity decrease is a very intriguing outcome. It is clearly visible that, under experimental conditions, at a relatively low compression, i.e., at the surface pressure of 10 mN m^−1^, CsA demonstrates a significantly better affinity for the PET_air_ substrate than DOPC.

The addition of the third component, LG, also greatly influences the intensities of the characteristic DOPC (Figure 5a) and CsA (Figure 5b,c) fragments in the group of the ternary DOPC-CsA-LG monolayers. Its presence in the ternary DOPC-CsA-LG 0.25 layer reduces the signal intensity of both CsA fragments: (C_6_H_14_N)^+^ (Figure 5b) and pseudomolecular ion (Figure 5c). This can be expected as a consequence of decreasing the molar fraction of CsA in the Langmuir monolayer. Moreover, it also suggests that the CsA orientation is maintained as the intensity of (C_6_H_14_N)^+^, as well as of the pseudomolecular ions, is reduced. A different, rather unexpected, situation occurs for DOPC-CsA-LG 0.50.

The intensity of (C_6_H_14_N)^+^ for DOPC-CsA-LG 0.50 is comparable to that observed for DOPC-CsA-LG 0.25 (Figure 5b), implying a similar amount of CsA molecules, while the intensity of the pseudomolecular ion is decreased (Figure 5c). As a similar amount of CsA molecules in the LB films for DOPC-CsA-LG with $\chi_{LG}$ equal to 0.25 and 0.50 is ruled out due to the different molar fraction of CsA, we hypothesize that with a smaller amount (surface coverage) of CsA (DOPC-CsA-LG 0.50), the molecules are more tilted towards the substrate which is favorable for a larger yield of (C_6_H_14_N)^+^ ion but unfavorable for the yield of the pseudomolecular ion. With greater tilting the CsA molecules are getting more perpendicularly oriented to the primary Bi^+^ beam, increasing the sputtering rate that leads to a greater fragmentation of the CsA molecules and a higher yield of (C_6_H_14_N)^+^. On the other hand, for DOPC-CsA-LG 0.75, the intensity of (C_6_H_14_N)^+^ is reduced while that of the pseudomolecular ion is similar to that for the ternary DOPC-CsA-LG 0.50. This suggests that the CsA molecules are less tilted (at LG molar fraction of 0.75) than they are in the ternary DOPC-CsA-LG 0.50, despite the smaller surface coverage with CsA (less amount of CsA molecules in the LB monolayer). The intensity of the pseudomolecular ion is similar to that observed for a larger surface coverage with CsA (DOPC-CsA-LG 0.50, Figure 5c). Furthermore, the lack of molecular ions of the single DOPC and LG in the mixed monolayers can confirm the existence of stronger attractive interactions between the molecules forming the multicomponent monolayers than between the single ones, which is in very good accordance with the magnitude of interactions revealed in the negative values of the Gibbs energy of mixing at the air/water interface [41]. Thus, the larger distance between the DOPC and DOPC as well as the LG and LG molecules in their monolayers the stronger the suppression of the yield of the molecular ions for these molecules.

Taking into account the above results, it can be strongly claimed that CsA does not interact chemically with DOPC and LG in the ternary monolayers. The interactions that occur between the CsA and DOPC and/or LG molecules in the LB films are of a physical nature (mainly H-bonds and Lifshitz–van der Waals forces) as was reported previously [41]. Moreover, it is possible that CsA changes its conformation from open to closed due to the hydrophobic environment provided by the hydrocarbon chains of DOPC (C18) and LG (C12). This is related to the formation of intramolecular hydrogen bonds in CsA molecules [64]. Such a mechanism (passive diffusion) can occur during drug transport through the lipid bilayer [65,66].

Figure 5d shows the distribution of the most characteristic fragments of LG, *m*/*z* = 153, 170, and 171, for the ternary DOPC-CsA-LG monolayers. No LG characteristic fragments for the pure LG layer were found. This means that DOPC and CsA play a primary role in the LG deposition, stabilization, and molecular arrangement on the PET_air_ substrate. Under the ultra-high vacuum conditions occurring inside the TOF-SIMS instrument, the deposited single LG monolayer can be removed from the PET_air_. This confirms that the monomolecular LG layer observed by AFM (Figure 3c) is weakly bound to the surface. On the other hand, the 4-fold increase in the intensity of LG fragments with *m*/*z* = 153 is seen as the LG molar fraction increases from 0.25 to 0.50 and from 0.50 to 0.75 in the ternary DOPC-CsA-LG films. A similar trend is noticeable for the other LG fragments (*m*/*z* = 170 and 171). This indicates that the increasing intensity of the most prominent ion of LG (*m*/*z* = 153) identified in the LB films on the PET_air_ substrate is not simply proportional to the increase in molar fraction in the mixed monolayers on the liquid subphase. This suggests that the yield of LG ions is closely dependent on the surrounding heteromolecules.

A similar effect is observed for DOPC (Figure 5a) where a reduction of the DOPC fragments’ intensity with a decreasing molar ratio of DOPC in the order: 0.375, 0.25, and 0.125, is expected. Surprisingly, the intensity of *m*/*z* = 104 and *m*/*z* = 166 fragments is only very slightly reduced, while that of the *m*/*z* = 184 fragment increases significantly. This indicates the involvement of the DOPC-LG interactions in the yielding intensity of all LG fragments in a similar way. The significant increase of *m*/*z* = 184 is determined by the specific location of LG towards the DOPC molecules that allows for the easier transfer of two hydrogen radicals from LG to DOPC.

Depending on the location of LG in relation to the DOPC molecule, the intensity of the fragments from the polar head group changes [58]. In a former paper it was shown that the intensity of *m*/*z* = 184 fragment was not increased when the LG was present in the mixed layer [59]. It was concluded that there are no direct interactions between the hydroxyl and phosphate groups. In the present study, the increasing intensity of the *m*/*z* = 184 fragment is observed for DOPC-CsA-LG 0.50 and DOPC-CsA-LG 0.75, while for DOPC-CsA-LG 0.25 it is slightly smaller than for DOPC-CsA 0.50 (Figure 5a). This demonstrates that at the smallest LG molar fraction (0.25) there are interactions between the hydroxyl groups of LG and the carbonyl groups of DOPC due to a smaller amount of LG, similar to that observed for the binary DOPC-LG mixture [58].

On the other hand, with the increasing molar fraction of LG (DOPC-CsA-LG 0.50 and DOPC-CsA-LG 0.75), direct interactions between the hydroxyl and phosphate groups of DOPC give rise to the intensity of the *m*/*z* = 184 fragment. The latter fact is possible due to the increasing ratio of LG to DOPC in the LB monolayer that allows the DOPC molecules to be surrounded by the LG ones. More detailed information about the exact location of the hydroxyl groups of LG towards the DOPC phosphocholine heads can be obtained from the distribution of 184/104 ratio and 184/166 ratio depicted in Figure 5e. At the smallest concentration of LG in the ternary DOPC-CsA-LG layer, the distribution of 184/104 ratio in comparison to 184/166 is similar to the single DOPC and binary DOPC-CsA monolayers. At a larger amount of LG in the monolayers, the ratio of 184/104 increases gradually. This may suggest the location of LG relative to the DOPC molecules. Namely, the hydroxyl groups of LG could be in a closer proximity to the oxygen atom bound with the phosphorus atom next to the hydrocarbon chains (a part of 184 ion) rather than the oxygen atom from the choline group (incorporated into the 104 ion) of DOPC, giving a larger yield of 184 and 166 fragments.

Moreover, the molar ratio of DOPC to LG at the air/water interface in the ternary DOPC-CsA-LG films is as follows: 1.50, 0.50, and 0.167 when the LG molar fraction increases in the order of 0.25, 0.50, and 0.75, respectively. This means that the DOPC to LG molar ratio decreases 3-times when the LG molar fraction increases from 0.25 to 0.50 and 0.50 to 0.75. Nearly the same values are observed for the molar ratio of DOPC to LG in the ternary DOPC-CsA-LG monolayers after their transfer onto the PET_air_ substrate (Figure 5f). This is because the intensity ratio of DOPC (*m*/*z* = 184) to LG (*m*/*z* = 170) decreases 2.81-times (34.73/12.33) when $\chi_{LG}$ increases from 0.25 to 0.50, and 2.93-times (12.33/4.20) when $\chi_{LG}$ increases from 0.50 to 0.75, respectively. The 184/153 ratio decreases 2.87-times and 2.96-times when the LG molar fraction changes from 0.25 to 0.50 and from 0.50 to 0.75. Such an excellent agreement proves unambiguously that the analogous molecular arrangement of the LG and DOPC molecules at the air/water interface and in the LB film occurs. Consequently, the molar ratio of LG to DOPC after the transfer on PET_air_ is maintained. This important finding demonstrates that the TOF-SIMS technique can be a useful tool, not only as a qualitative but also a quantitative method, in the surface chemistry analysis of monolayers.

In order to evaluate the potential interactions between the DOPC and CsA molecules in the examined monolayers, the binary DOPC-CsA monomolecular films with different molar fractions of CsA ($\chi_{CsA}$ = 0.25, 0.50, 0.75) were deposited onto the PET_air_ substrate. Figure 6 shows the distribution of the intensity of the most characteristic fragments identified in the positive TOF-SIMS spectra.

The intensity of all DOPC fragments decreases about 3-times when the CsA molar fraction increases from 0.25 to 0.50 and subsequently from 0.50 to 0.75 (Figure 6a). This behavior is different from that observed for the ternary DOPC-CsA-LG monolayers. The surface coverage with CsA (fragment (C_6_H_14_N)^+^, Figure 6b) and ordering (pseudomolecular ion, Figure 6c) demonstrate the highest intensity values for the DOPC-CsA 0.50 film. This proves that, in this mixed layer, the CsA molecules are the most vertically oriented towards the substrate. It is determined by the efficient repulsion of CsA by the DOPC molecules [41] which in consequence, changes the orientation/tilting of CsA molecules. Moreover, at the greatest CsA molar fraction (0.75) a smaller intensity of the (C_6_H_14_N)^+^ fragment can suggest a smaller surface coverage with CsA in relation to the DOPC-CsA 0.50 monolayer. This intriguing observation implies that the DOPC-CsA 0.75 monolayer is not transferred quantitatively from the liquid phase to the PET_air_ substrate. Moreover, a significantly greater reduction in the CsA pseudomolecular ion (Figure 6c) intensity than that of (C_6_H_14_N)^+^ (Figure 6b) indicates that the CsA molecules are more tilted than is observed for DOPC-CsA 0.50. Indeed, CsA could be not fully quantitatively transferred on the PET_air_ substrate. The above results show that the DOPC molecules play an important role during the transference of CsA. For the optimal composition (DOPC-CsA 0.50) the DOPC molecules can fill the gaps efficiently outside and inside the CsA molecules. Consequently, the largest surface coverage with CsA and ordering (more vertical orientation) occur. With the minimal amount of DOPC (DOPC-CsA 0.75), the CsA molecules are very likely to repel each other, reducing the surface coverage and the area of the continuous monolayer, as is proven by the smaller pseudomolecular ion intensity (Figure 6c). Moreover, distribution of the intensity ratio of the DOPC fragments (Figure 6d) suggests that there is not a hydrogen radical transfer from the CsA to the DOPC molecules, as is observed for the interactions occurring between the DOPC and LG molecules. The 184/104 and 184/166 ratio intensities demonstrate the similar values for DOPC-CsA 0.25 and DOPC-CsA 0.50. It can be determined by the fact that at the smaller surface coverage with DOPC (DOPC-CsA 0.50, at the larger amount of CsA) the hydrogen radical ions can be supplied by the surrounding DOPC molecules due to their greater proximity. In the DOPC-CsA 0.75 monolayer, the distance between the molecules is larger, thus reducing the number of available hydrogen radicals and increasing the yield of the *m*/*z* = 184 fragment. Overall, the above results demonstrate the efficient force of repulsion between the CsA and DOPC molecules that changes the CsA orientation significantly. As was mentioned before, DOPC plays an important role in the transferring and ordering of the CsA molecules.

Based on these results one can hypothesize that CsA is preferentially adsorbed from the binary DOPC-CsA monolayer onto the activated solid substrate which determines the surface properties of the DOPC-CsA monomolecular film, revealed also in the values of the contact angles (see Section 3.4).

The TOF-SIMS analysis enabled an estimation of the arrangement of the molecules forming mixed monolayers. The experiment proved that LG in the ternary DOPC-CA-LG monolayer with $\chi_{LG}$ = 0.25 is localized close to the oxygen atom bound with phosphorus next to the hydrocarbon chain (*m*/*z* = 184) of DOPC. By increasing its molar fraction, LG can penetrate more deeply into the DOPC polar head region. In addition, the DOPC and LG molecules can be located outside as well as inside the CsA molecules.

### 3.4. Contact Angles (CA) and Surface Free Energy (SFE)

In the next stage of the experiments, the wettability of the activated by air low temperature plasma PET substrate and modified with single (DOPC, CsA, LG), binary (DOPC-CsA 0.50) and ternary (DOPC-CsA-LG) monolayers of different molar fractions of LG, were tested by the water, formamide, and diiodomethane contact angle (CA) measurements. Then, based on the CA values, the surface free energy (SFE) and its components were estimated by applying the LWAB model (Equations (3)–(5)). The selection of the three liquids used to determine these parameters is a controversial issue. Depending on their use, different values of the energy components can be obtained. For verification, Jańczuk et al. studied various combinations of five liquids: water, formamide, glycerol, diiodomethane, and bromoform [67]. As a result of the measurements of contact angles on the cholesterol and adsorbed bile salts surfaces, they found that the optimal trio of liquids should consist of one apolar liquid with a relatively high surface tension (diiodomethane—D) and two polar liquids with significantly different surface tension components (water—W and formamide—F). The use of such a trio for the contact angle measurements results in the smallest error in determining the energy components, thus providing the most reliable results. On the other hand, two weakly monopolar liquids (diiodomethane and bromoform) or two polar liquids with similar surface tension components (formamide and glycerol) should not be included in the set of utilized liquids due to significant deviations in the calculation of the energy components. Based on the above data, we exploited the W-F-D trio in our research to determine the values of the surface free energy and its components, which are apparently dependent on the type of used liquids.

An examination of the wetting properties of the modified PET substrates is very important because it gives more profound insight into understanding the interactions between the molecules of the test liquids and the solid surface as well as the influence of film composition on the type of interactions. Liquids of a different polarity can reflect environments of a different nature.

**Figure 7:**
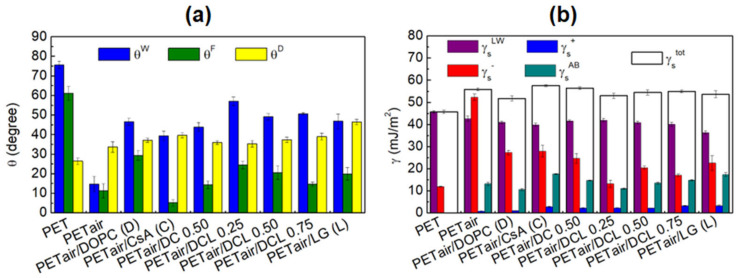
Average advancing contact angles of water, $\theta^{W}$, formamide, $\theta^{F}$, and diiodomethane, $\theta^{D}$, measured onto the unmodified and modified PET surfaces (**a**) and total surface free energy and its components calculated from the LWAB approach (**b**) for the unmodified and modified PET surfaces.

Figure 7 shows the obtained values of the average advancing contact angles of the three test liquids (Figure 7a), and the total surface free energy and its components (Figure 7b). As mentioned previously, the unmodified PET substrate has a hydrophobic character [53,68,69] which is reflected in the values of contact angles. The contact angles of the two polar liquids are quite high, the water contact angle, $\theta^{W}$ is equal to 75.6° ± 1.9°, while that of formamide, $\theta^{F}$ = 61° ± 3.5° (Figure 7a). The contact angle of apolar diiodomethane, $\theta^{D}$ is equal to 26.4° ± 1.7° (Figure 7a). The values of these contact angles correspond to the low value of the total surface free energy, $\gamma_{s}^{tot}$ = 45.6 mJ m^−2^, as calculated based on Equations (4) and (5). The total SFE in that case is equal to the Lifshitz–van der Waals component ($\gamma_{s}^{LW}$). The air plasma action (PET_air_) changes the wetting properties of the PET surface significantly. Both $\theta^{W}$ and $\theta^{F}$ decrease, and $\theta^{D}$ increases (Figure 7a). Thus, the increase in the total SFE is also observed, $\gamma_{s}^{tot}$ = 55.8 mJ m^−2^, which can be correlated with the increased $\gamma_{s}^{-}$ forces and the occurring acid–base interactions ($\gamma_{s}^{AB}$ = 13.2 mJ m^−2^). These observations and the increase in the surface roughness parameter (Figure 3b) confirm that the plasma action introduces new functional groups containing oxygen and nitrogen (-OH, C-O, O=C-O, C=O, N-CO-N) to the PET substrate [51] so that the interactions between the surface and the liquid molecules take place by hydrogen bonds. This is evidenced by the enhanced electron-donor parameter. On the other hand, the interactions by the π-electrons included in the Lewis–base interactions are possible due to the presence of the PET_air_ aromatic rings. Thus, the coating process can be conducted efficiently which was confirmed by other authors in their experiments [10].

To exclude possible reorganization of the monolayers during their contact with the liquids, additional measurements of the contact angle as a function of time were conducted for the single DOPC monolayer deposited on the PET_air_. The advancing contact angles of the three test liquids were measured within 60 s of being deposited onto the PET_air_/DOPC substrate. The obtained results are presented in Table 3.

As can be seen, the contact angle values are practically constant within the first 10 s. There are only minor variations, particularly in the case of the diiodomethane contact angle. This is related to the dispersion interactions between the monolayer molecules and this apolar liquid. For the polar liquids (water and formamide) greater fluctuations in the contact angle values are noticeable. The above experiments show that the LB film is stable during the contact angle measurements performed immediately (within the first few seconds) after placing a drop of the test liquid onto the analyzed surface. In this way, the measured value of the contact angle determines the real energy state of the monolayer, which relates to its original structure.

Deposition of the monolayers onto the activated by air low temperature plasma PET substrate causes the increase of contact angle values in comparison with PET_air_, but a decrease concerning the unmodified PET. The presence of the deposited monolayers onto the polymer surface was confirmed by the AFM (Figure 3 and Figure 4) and TOF-SIMS analyses (Figure 5 and Figure 6).

**Table 3:** Average advancing contact angles of the test liquids measured on the single DOPC monolayer deposited on PET_air_ during 60 s.

Time (s)	Average Advancing Contact Angle		
	Water	Formamide	Diiodomethane
1	49.3 ± 0.5	31.3 ± 2.1	40.4 ± 0.6
2	49.2 ± 0.4	31.0 ± 1.9	40.4 ± 0.7
3	49.1 ± 0.4	30.9 ± 1.9	40.2 ± 1.0
4	49.3 ±0.4	30.9 ± 2.0	40.4 ± 0.7
5	49.4 ± 0.4	30.7 ± 2.2	40.2 ± 1.0
6	49.2 ± 0.2	30.5 ± 2.4	40.2 ± 1.0
7	49.1 ± 0.5	30.4 ± 2.4	40.2 ± 1.0
8	49.0 ± 0.4	30.3 ± 2.3	40.2 ± 1.0
9	49.2 ± 0.7	30.2 ± 2.5	40.2 ± 1.0
10	49.1 ± 0.5	30.2 ± 2.4	40.2 ± 1.0
20	48.8 ± 0.5	29.5 ± 2.6	40.2 ± 1.0
30	48.1 ± 0.8	29.2 ± 2.7	40.2 ± 0.9
40	47.9 ± 0.4	29.1 ± 2.7	40.0 ± 1.0
50	47.1 ± 0.2	29.1 ± 2.8	40.0 ± 1.0
60	47.1 ± 0.1	29.1 ± 2.8	40.0 ± 1.0

The presence of a single DOPC monolayer results in the increase of the contact angle for all three test liquids (Figure 7a: $\theta^{W}$ from 14.7° ± 3.9° to 46.6° ± 1.8°, $\theta^{F}$, from 11.3° ± 3.6° to 29.3° ± 2.4°, and $\theta^{D}$ from 33.7° ± 2.7° to 37.1° ± 1.1°). Thus, the total value of the surface free energy ($\gamma_{s}^{tot}$ = 51.7 mJ m^−2^, Figure 7b) and its components decreases. After the DOPC deposition the contribution of the $\gamma_{s}^{-}$ to the interactions between the liquid molecules and the monolayer is almost twice lower, while the $\gamma_{s}^{AB}$ decreases slightly. The higher contact angle and the lower total SFE values indicate that the surface has a weaker polar character. This can be correlated with the specific organization of the DOPC molecules where the polar head groups are closer to the activated by air plasma PET substrate, and the hydrocarbon chains are located outwards, and tilted towards the solid substrate, which is more accessible to the liquid molecules. The tilting of the DOPC molecules was affirmed by the lack of molecular ions in the previously presented mass spectra (Section 3.3). The single CsA layer deposited on PET_air_ shows a greater polarity than DOPC (lower values of $\theta^{W}$ and $\theta^{F}$, Figure 7a). Conversely, $\theta^{D}$ increases (in comparison to PET_air_/DOPC, Figure 7a).

The changes in the hydrophobic-hydrophilic character are also visible in the total surface free energy and its components. The biggest one is observed for $\gamma_{s}^{AB}$ (Figure 7b) as a result of the particular increase in the electron-donor interactions originating from the added by plasma action functional groups capable of acid–base interactions. The $\gamma_{s}^{-}$ parameter decreases confirming that the functional groups of the PET_air_ are involved in the interactions with the CsA polar groups. Moreover, the surface free energy changes are also related to the specific molecular structure of CsA, where the part of the polar groups connected to the aminoacid ring is located near the PET_air_, and the rest towards the air. These moieties located outwards could interact with the liquid molecules. Furthermore, the conformation of CsA molecules depends on the polarity of their environment [65,66]. Accordingly, the transfer of CsA from the aqueous subphase to the activated PET with properties other than water, thereby with different interactions, may provoke changes in its molecular conformation and monolayer organization. Therefore, some aggregates can be observed (Figure 3e).

Mixing the CsA and DOPC molecules to form a binary monolayer induces the increase in the $\gamma_{s}^{tot}$ (Figure 7b) value (from 51.7 mJ m^−2^ to 56.3 mJ m^−2^) but does not have a large impact on the contact angle values (Figure 7a) at the same time, in relation to the single DOPC layer. The biggest one is observed for the $\theta^{F}$ value, while $\theta^{W}$ and $\theta^{D}$ are almost the same (Figure 7a). The change in the $\gamma_{s}^{tot}$ is related to a small decrease in the electron-donor ($\gamma_{s}^{-}$) and an increase in the acid–base ($\gamma_{s}^{AB}$) interactions. As was proven by the mass spectrometry, repulsion between the DOPC and CsA molecules induces the CsA orientation changes to a more vertical one (the biggest intensity of the molecular ions, Figure 6c). As a result, the liquids, especially formamide, can interact more easily with the PET_air_ substrate. Moreover, formamide has the amino and carbonyl groups in its structure which have the ability to interact with the CsA molecule by the H-bonds.

The addition of the LG molecules results in significant changes in the surface polarity. For all ternary layers, the decrease in $\gamma_{s}^{LW}$ and $\gamma_{s}^{-}$, as well as the increase in the $\gamma_{s}^{+}$ values are observed (Figure 7b). This suggests that the presence of the LG molecules in the ternary layers weakens the electron-donor while enhancing the electron-acceptor interactions between the monolayer components and molecules of the test liquids. Additionally, occurrence of the LG in the monolayers increases the acid-base interactions ($\gamma_{s}^{AB}$) with the liquids. This is related to the structure of the antioxidant, where the π-π interactions can occur between the aromatic ring [70] and liquid molecules. The biggest value of $\theta^{W}$ (57.1° ± 2.2°), $\theta^{F}$ (24.4° ± 2.0°), and the smallest $\theta^{D}$ (35.3° ± 1.6°) contact angles are obtained (Figure 7a) for the ternary DOPC-CsA-LG 0.25 film. Then, the greater molar fraction of LG in the monolayer, the smaller contact angles are gained for the polar liquids and bigger for the apolar—diiodomethane. The smallest value of $\theta^{D}$ is measured for DOPC-CsA-LG 0.50 (49.1° ± 1.6°). This could be related to the strongest interactions, and tight packing of molecules in the monolayer where the hydrocarbon chains are located, towards the test liquids, while the polar headgroups are positioned to the solid surface and/or are inaccessible [41]. The water contact angle for DOPC-CsA-LG 0.75 is slightly smaller (50.6° ± 0.6°) while the highest value of $\theta^{D}$ (39.0° ± 1.8°) is acquired. These CA values (Figure 7a) have a direct impact on the surface free energy and it components’ values (Figure 7b). A very similar value of the total SFE is observed for the ternary DOPC-CsA-LG 0.50 and DOPC-CsA-LG 0.75, ($\gamma_{s}^{tot}$ equals 54.4 mJ m^−2^ and 54.9 mJ m^−2^, respectively). The main differences in the type of interactions between these two monolayers and liquid molecules are manifested through the $\gamma_{s}^{-}$ and $\gamma_{s}^{+}$ changes. For the DOPC-CsA-LG 0.50 thin film the electron-donor parameter ($\gamma_{s}^{-}$) is quite a bit higher (by 3.39 mJ m^−2^) while the electron-acceptor parameter ($\gamma_{s}^{+}$) is lower (by 1.9 mJ m^−2^, Figure 7b).

The single LG layer is characterized by the smaller surface free energy than all three ternary DOPC-CsA-LG monolayers ($\gamma_{s}^{tot}$ = 53.6 mJ m^−2^). Additionally, $\gamma_{s}^{-}$ and $\gamma_{s}^{AB}$ calculated based on the contact angle values are higher than for the ternary coatings, ($\gamma_{s}^{-}$ = 22.6 mJ m^−2^ and $\gamma_{s}^{AB}$ = 17.4 mJ m^−2^). This fact confirms the enhanced contribution of the electron-donor and π-π interactions between the LG and liquids molecules. Moreover, the π-π interactions can determine the monolayer ordering such that the aryl ring can interact with four other adjacent aryl groups [71]. Similar conclusions were drawn by Huang et al. The researchers reported that both π-π and hydrogen interactions had a significant impact on the organization of the polydopamine (PDA) monolayer [72].

As was mentioned in the Introduction section, hybrid coatings can be used in drug delivery systems as well as tissue engineering including implantology. The examination of the hydrophobic-hydrophilic character by contact angle measurements, as well as a calculation of the total surface free energy and its components, is a key point for selecting the appropriate composition of coating to obtain a surface with desired properties. The hydrophobic-hydrophilic character, as well as the topography of the implant surface which are in contact with the blood cells, greatly influence the immune response of the host body [73,74]. The conducted wettability experiments prove that the ternary DOPC-CsA-LG monolayers are less hydrophilic than the binary DOPC-CsA 0.50 and all the single (DOPC, CsA, LG) layers. [41]. DOPC has *cis*-double bonds in its structure while LG has a quite big polar head in comparison with the hydrophobic chain. In turn, the cyclosporine molecules have a large aminoacid ring whereby liquids can gain and interact with the PET_air_ substrate, not only with the monolayer molecules. All these factors make it possible for the test liquid molecules to reach the substrate. Thus, both the DOPC and LG molecules can not form largely packed monolayers as they are tilted towards the PET_air_ surface. The smaller hydrophilicity of the ternary monolayers is related to the strong attractive interactions between the molecules forming films. Therefore, more compact, homogeneous thin films are produced. As a result, the liquid molecules can not reach the activated PET substrate, creating larger contact angles. Based on these findings all three-component layers have great application potential for implants’ coating. In the group of the ternary monolayers, the DOPC-CsA-LG 0.50 film is characterized by the best wetting properties which is related to the strongest attractive interactions between the molecules forming the monolayer [41].

## 4. Conclusions

In this paper the Langmuir–Blodgett technique was used to transfer the single (DOPC, CsA, LG), binary (DOPC-CsA 0.50), and ternary DOPC-CsA-LG ($\chi_{LG}$ = 0.25, 0.50, 0.75) monolayers from the water subphase onto the activated by air plasma PET substrate (PET_air_). For the physicochemical characterization of the tested monolayers, the Brewster angle microscopy (BAM), atomic force microscopy (AFM), time-of-flight mass spectrometry (TOF-SIMS), and contact angle measurements (CA) were employed. The results obtained by means of these techniques confirmed the effective transfer of all monolayers to the PET_air_ substrate. The surface study in the nanoscale affirmed that during the PET activation with the low temperature air plasma, new functional groups are added. This was evidenced by the increasing roughness ($S_{q}$ parameter) and SFE, in relation to the unmodified PET. On the other hand, after the thin film’s deposition on PET_air_, its roughness decreased. Among the single monolayers, the smoothest surface was obtained for the LG monolayer. The three-component DOPC-CsA-LG 0.50 monomolecular layer was characterized by the presence of domains similar to those observed for the single DOPC monolayer, which was confirmed by the obtained profiles (Figure 4d,g). In the case of the lower and higher lauryl gallate fractions ($\chi_{LG}$ = 0.25 and 0.75) in the layer, these domains were absent. The surface morphology was strongly associated with the transfer ratio values (Table 1).

The use of mass spectrometry allowed for the determination of the arrangement of the molecules forming the monolayers. Thus, it was confirmed that the LG molecules locate beside the DOPC molecules, penetrating more deeply into the DOPC polar head group region when the lauryl gallate molar fraction in the layer increases. The presence of the two C18 (DOPC) and one C12 (LG) chains makes the environment hydrophobic. Thus, the CsA molecule can change its conformation from an open to closed one. Therefore, the interactions between the monolayer molecules have dispersive nature. The TOF-SIMS analysis also showed a great correlation between the qualitative and quantitative composition of the monolayers at the air/water interface and on the solid support.

The contact angle measurements determined the total surface free energy and its components, allowing for the evaluation of the wetting properties of the modified PET substrates. Among all mixed monolayers, the ternary DOPC-CsA-LG films with the LG molar fractions of 0.50 and 0.75 are characterized by the highest value of surface free energy.

All research techniques confirm the possibility of the application of the Langmuir monolayers, including the phospholipid DOPC, the immunosuppressant CsA, and the antioxidant LG as potential biocompatible coatings for, e.g., stents used in tissue engineering. Among them the most promising ones seem to be the DOPC-CsA-LG 0.50 and DOPC-CsA-LG 0.75 monolayers.

## Data Availability

Not applicable.
